# Identification and quantification of VOCs by proton transfer reaction time of flight mass spectrometry: An experimental workflow for the optimization of specificity, sensitivity, and accuracy

**DOI:** 10.1002/jms.4063

**Published:** 2018-02-21

**Authors:** Andrea Romano, George B. Hanna

**Affiliations:** ^1^ Department Surgery and Cancer Imperial College London UK

**Keywords:** breath analysis, ion chemistry, method development, PTR‐ToF‐MS, volatile organic compounds

## Abstract

Proton transfer reaction time of flight mass spectrometry (PTR‐ToF‐MS) is a direct injection MS technique, allowing for the sensitive and real‐time detection, identification, and quantification of volatile organic compounds. When aiming to employ PTR‐ToF‐MS for targeted volatile organic compound analysis, some methodological questions must be addressed, such as the need to correctly identify product ions, or evaluating the quantitation accuracy. This work proposes a workflow for PTR‐ToF‐MS method development, addressing the main issues affecting the reliable identification and quantification of target compounds. We determined the fragmentation patterns of 13 selected compounds (aldehydes, fatty acids, phenols). Experiments were conducted under breath‐relevant conditions (100% humid air), and within an extended range of reduced electric field values (E/N = 48–144 Td), obtained by changing drift tube voltage. Reactivity was inspected using H_3_O^+^, NO^+^, and O_2_^+^ as primary ions. The results show that a relatively low (<90 Td) E/N often permits to reduce fragmentation enhancing sensitivity and identification capabilities, particularly in the case of aldehydes using NO^+^, where a 4‐fold increase in sensitivity is obtained by means of drift voltage reduction. We developed a novel calibration methodology, relying on diffusion tubes used as gravimetric standards. For each of the tested compounds, it was possible to define suitable conditions whereby experimental error, defined as difference between gravimetric measurements and calculated concentrations, was 8% or lower.

## 1 INTRODUCTION

Proton transfer reaction mass spectrometry (PTR‐ToF‐MS) is a direct injection MS technique, which is widely employed in the analysis of volatile organic compounds (VOCs); its main fields of application are environmental research, food analysis, medicine, and homeland security.^1^

PTR‐ToF‐MS commercial instruments offer limits of detection in the low pptV range (pptV = parts per trillion in volume) and a linear range spanning over more than 6 orders of magnitude.^2^, ^3^ PTR‐ToF‐MS in its original configuration was based exclusively on chemical ionization by proton transfer. With the introduction of the selective reagent ion add‐on, alternative forms of chemical ionization became possible by using NO^+^ and O_2_^+^ as primary ions.^4^ Compound identification heavily relies on the measurement of mass‐to‐charge ratios, which is accurate up to the second or third decimal digit, depending on calibration accuracy and performance of the mass analyzer. For a reliable identification of the parent compounds starting from mass spectral data, ionization should be “soft,” with ideally 1 single product ion generated for every parent compound and no fragmentation. Fragmentation is largely dependent upon reduced electric field within the drift tube. This parameter is conveniently summarized by the E/N parameter, which can be easily modified by changing drift voltage, temperature, or pressure. Lower E/N values will result in a reduction in drift velocity and fragmentation, with increased sensitivity. Under the ionization conditions most commonly employed in PTR‐MS (E/*N* = 120–150 Td, 1 Td = 10^−17^ V cm^2^), high fragmentation can sometimes compromise the detection and identification of some compounds. This is well exemplified by aldehydes: when using H_3_O^+^ as primary ion, dissociative reaction channels prevail and the relative abundance of the protonated ion [M + H]^+^ is as low as 6% to 9% with C5‐C8 compounds.^5^ To date, even though the potential benefits of working under conditions of low reduced drift field are theoretically known, the actual exploration of such conditions is limited. The reactivity of several VOCs (mostly alkanes) was studied employing NO^+^ as primary ion at 60 Td E/N.^6^ The possibility of employing similar conditions for H_3_O^+^ was demonstrated, provided cluster chemistry and adduct formation are taken into account.^7^ The use of O_2_^+^ as primary ion under low reduced drift field conditions has, to our knowledge, never been reported.

Several techniques are nowadays available for calibration of a VOC measurement. The most commonly adopted approach relies on gas calibration mixtures.^8^ The main limitation lies in the cost and reduced availability of VOCs as diluted calibration gases. The use of dynamic solution injection (DSI) obviates the problem of sample availability: in DSI, a solution containing the compounds of interest is vaporized into a heated chamber and injected into the instrument.^9^ The main disadvantage of DSI lies in the need to find a suitable medium where to dissolve the analytes of interest: water is ideally suited for PTR‐MS analysis, but it will not dissolve non‐polar VOCs, whereas the use of most organic solvents or co‐solvents will saturate the detector. Permeation or diffusion tubes represent the most straightforward approach^10^: a permeation/diffusion device can be prepared using any available compound, regardless of solubility. The VOC concentration in the gas phase can be varied by altering the device equilibration temperature and/or flow. Most importantly, by measuring analyte losses at the end of the experiment, it is possible to standardize the results against a gravimetric measurement.

The aim of the work presented in this manuscript was to describe an experimental workflow that helps researchers to set up the appropriate methods for optimum sensitivity, specificity, and accuracy of VOC measurements. The 13 compounds chosen for this study belong to 3 chemical classes (aldehydes, fatty acids, and phenols). Fragmentation patterns were determined at different E/N values under breath‐relevant conditions. The impact of water vapor on fragmentation was assessed, and analytical accuracy was determined by comparison with gravimetric standards. The compounds examined in the work were previously found to be elevated in the exhaled breath of patients suffering from oesophago‐gastric adenocarcinoma.^11^ These results were obtained using another established technique for VOC analysis: selected ion flow tube mass spectrometry (SIFT‐MS).^12^ The present work also provides interesting insight regarding differences and analogies between PTR‐MS and SIFT‐MS chemistry.

## 2 MATERIALS AND METHODS

### 2.1 Materials

All chemical standards were purchased from Sigma Aldrich (St. Louis, MO) and were of the highest available purity grade. The compounds employed included 7 aliphatic linear aldehydes (butanal, pentanal, hexanal, heptanal, octanal, nonanal, decanal), 3 short‐chain fatty acids (butanoic acid, pentanoic acid, hexanoic acid), and 3 phenols (phenol, 4‐methyl‐phenol, and 4‐ethyl‐phenol).

### 2.2 Fragmentation pattern determination

For the determination of the fragmentation patterns, we adapted a procedure described in the literature.^13^ Individual VOC solutions were prepared in de‐ionized water at concentrations ranging from 0.03 to 1% (*v*/v) for the aldehydes and fatty acids and at 100 mg/L for the phenols. A 500‐mL glass bottle, equipped with a Drechsel head, was filled with 100‐mL de‐ionized water and kept in a water bath at 37°C. A flow of clean air was generated by means of a pump, connected to a hydrocarbon trap (Supelco Supelpure HC, Sigma Aldrich). An airflow of approximately 500 sccm was applied to the bottle. Measurements were conducted employing a commercial PTR‐MS instrument (PTR‐TOF 1000, Ionicon Analytik GmbH, Innsbruck, Austria). At the beginning of the measurement, drift tube conditions were: temperature 110°C, pressure 2.30 mbar, and voltage 600 V, resulting in an E/N of 144 Td (1 Townsend = 10^−17^ V cm^2^). The PTR‐MS inlet was connected to the bottle head with a flow of 40 sccm, letting the excess flow out by means of a t‐piece. The inlet consisted of a PEEK tubing heated at 110°C. Once temperature and signal were stable, 1 mL of standard solution was added to the bottle by means of a syringe: several mass peak traces rapidly increased following addition and subsequently decreased, showing a time evolution pattern that was characteristic for each parent compound. This allowed to attribute unambiguously, for each compound and set of conditions, parent ion, and relative fragments or adducts. After standard solution addition and the initial equilibration phase, E/N was decreased in a stepwise fashion, with 1‐min steps. The voltage applied were as follows: 600, 550, 500, 450, 400, 350, 300, and 200 V, resulting in *E*/*N* = 144, 132, 120, 108, 96, 84, 72, and 48 Td, respectively. At the end of each experiment, the reagent ion was switched, and a new measurement was started using a fresh bottle, employing the same protocol and standard solution.

The effect of the reduction in air water content on fragmentation patterns was studied by employing the same experimental setup. The measurement was started using water‐saturated air and setting the instrument drift voltage at 350 V. Once steady conditions were reached, the switch to dry conditions were achieved by connecting a CaCl_2_ cartridge in between the Drechsel bottle and the PTR‐MS inlet. The cartridge consisted of a 10‐mL glass vial equipped with a PTFE septum, filled with anhydrous calcium chloride (CaCl_2_, Sigma‐Aldrich). After cartridge connection, approximately 30 seconds were required to re‐establish steady conditions. Since the volume of the cartridge was relatively low compared with the inlet flow, no measurable retention of analytes onto the cartridge could be observed. Measurement was then continued for approximately 1 minute, allowing to establish fragmentation patterns under dry conditions.

### 2.3 Comparison with gravimetric measurement

Diffusion tubes were prepared for all standards. They consisted of a 2‐mL glass vial equipped with a PTFE septum, pierced by means of a PEEK tubing piece. By changing length and inner diameter of the tubing, it was possible to modify the release rate of each diffusion tube. In order to maintain a constant rate of VOC release, we employed a permeation unit (ES 4050P, Eco Scientific, Stroud, Gloucestershire UK). The unit oven was set at 60°C and kept under a constant flow of nitrogen (2000 sccm). Each measurement was carried out for 12 to 16 hours, filling the oven with different diffusion tubes and cycling the PTR‐MS source with the 3 primary ions in a continuous loop (5 min/reagent ion). Ionization conditions were: 110°C, 2.3 mbar, and 350 V, resulting in 84 Td E/N.

VOC concentrations were estimated applying the well‐established theory.^14^ When using H_3_O^+^ as primary ion, water clusters are negligible for E/N values above 120 Td, while at lower reduced electric field values, the hydronium cluster (H_2_O) H_3_O^+^ increases above 10% of the total primary ion counts. The formula for concentration calculation needs thus to be modified considering cluster reactivity, also following previous recommendations.^7^ To overcome primary ion signal saturation, hydronium ion and cluster signals were extrapolated from the corresponding ^18^O isotopologues, at *m/z* 21.02 and 39.03 Th, respectively. When employing NO^+^ as primary ion the formation of hydrated nitrosonium ion clusters (H_2_O) NO^+^ remained below 5% and its contribution to ionization was thus not considered. To overcome mass peak saturation, the NO^+^ signal was extrapolated from the corresponding ^15^N isotopologue at m/z 30.99 Th. In the case of ionization using O_2_^+^, the signal was extrapolated from the corresponding mono‐atomic ^18^O^16^O isotopologue (m/z 33.99 Th). Ion cluster formation was negligible (<1% of total signal).

All raw signals were modified correcting for the detector‐specific mass discrimination properties, by establishing the so‐called “transmission curve.” This was obtained by measuring a certified mixture of aromatic hydrocarbons, all at the concentration of 100 ppbV (TO‐14 Aromatics Subset Mix, Sigma Aldrich). The transmission values were established by interpolating the measured intensity values of reference mass peaks by means of a natural spline. Reaction rate constants were calculated using Su's parametrization approach.^15^ This allows to consider non‐thermal conditions typical of commercial PTR‐MS instruments, allowing for more accurate quantification.^16^ All reaction rate constants and the parameters used to calculate them are reported in Table S1 (Supplementary Material).

### 2.4 Quality control

During the described experiments, a series of quality checks were conducted on the PTR‐ToF‐MS daily. Impurities with the 3 ionization modes were O_2_^+^ (<2%) for H_3_O^+^ as primary ion, NO_2_^+^ (<3%) for O_2_^+^ as primary ion, and NO^+^ and NO_2_^+^ (< 5% altogether) for O_2_^+^ as primary ion, respectively. Quantitation accuracy with the 3 ions was within ±10% of a certified standard, represented by a Trace Source™ benzene permeation tube (Kin‐Tek Analytical Inc., La Marque TX). The consistency of fragmentation with NO^+^ and H_3_O^+^ as primary ions was assessed by measuring the ratio between reference mass peaks with given standard compounds. For NO^+^, we used the ratio between peaks *m/z* 71 and 43 using a butanal permeation tube standard. For H_3_O^+^, we used the ratio between peaks *m/z* 89 and 71 using a butanoic acid permeation tube standard. The values measured on the different days were within ±2% of the mean. Whenever required, we optimized the voltage of the microchannel plate and the mass resolution (>1500 m/Δm), using *m/z* 89 (butanoic acid with H_3_O^+^) as reference peak.

### 2.5 Data analysis

Data were extracted using PTRMS viewer version 3.2.2.2 (Ionicon Analytik). Additional data analysis was conducted using in‐house generated scripts written using R programming language.^17^

## 3 RESULTS AND DISCUSSION

### 3.1 Aldehyde reactivity using NO^+^ as primary ion

**Figure 1:**
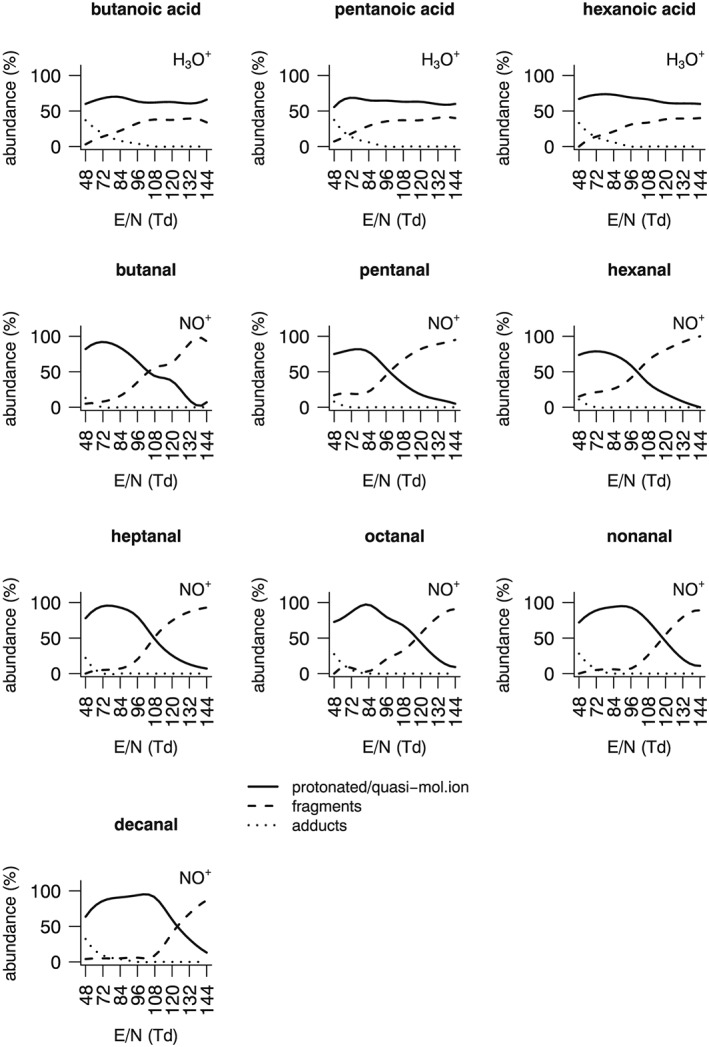
Fragmentation patterns of fatty acids and aldehydes in humid air using H_3_O^+^ and NO^+^ as primary ions, respectively

Fragmentation patterns were determined under humid air conditions for all linear saturated aldehydes containing between 4 and 10 carbon atoms. As previously reported for PTR‐MS,^4^ when using NO^+^ as primary ion, aldehydes undergo hydride abstraction producing quasi‐molecular ions having nominal mass [M − H]^+^. This reaction channel is of interest for aldehyde analysis, as it allows to distinguish them from isomeric ketones, having different reactivity. Ideal reaction conditions would be those where the only reaction product is the quasi‐molecular ion and no fragments or adducts are generated, thus allowing for maximum sensitivity and specificity, with minimum product ion overlaps. Aldehyde reactivity under different collision energy conditions is shown in detail in the case of decanal (Table 1). When operating within the reduced electric field range most commonly employed in PTR‐MS analysis (E/*N* = 120–144 Td), ionization often proceeded with fragmentation and generation of alkyl fragments. At lower reduced electric field values (E/*N* = 96–108 Td), most fragmentation reactions were suppressed, and only products at m/z = 137 and 155 were observed, representing the products of water loss and hydride abstraction, respectively. When using E/N values below 96 Td, limited water adduct formation was observed along with the previously mentioned [M − H]^+^ ion. All tested aldehydes showed similar behavior (Figure 1). Optimal conditions were observed in the range 72 to 96 Td, where the quasi‐molecular ion amounted to 78%‐97% of the total product ions obtained. Longer‐chain aldehydes showed an increased tendency to form water adducts at low E/N values, whereas shorter‐chain aldehydes displayed increased fragmentation under more energetic conditions. The observed reactivity was in overall good agreement with previous reports,^18^ where aldehyde reactivity was only described under high reduced electric field conditions (E/N = 130 Td). PTR‐MS and SIFT‐MS show overall similarities in the capability to allow for aldehyde determination by using hydride transfer as main reaction channel. Unlike PTR‐MS, aldehyde ionization in SIFT‐MS proceeds without fragmentation, but with the generation of variable (4%–26%) amounts of [M + NO]^+^ adducts.^19^

**Table 1:** Fragmentation patterns at different E/N values (expressed in Td) obtained for a few selected compound/primary ion combinations

					% Relative Abundance							
Compound	Primary Ion	Meas. Mass (Th)	Theor. Mass (Th)	Proposed Product and Reaction Channel	E/N 144	E/N 132	E/N 120	E/N 108	E/N 96	E/N 84	E/N 72	E/N 48
Hexanoic acid	H_3_O^+^	135.09	135.10	C_6_H_12_O_2_ + H_2_O + H_3_O^+^ → **C**_**6**_ **H**_**12**_ **O**_**2**_ **·H**_**2**_ **O** ^**+**^+ H_2_O	−	−	−	−	−	6	13	33
		117.09	117.09	C_6_H_12_O_2_ + H_3_O^+^ → **C**_**6**_ **H**_**13**_ **O**_**2**_^**+**^+ H_2_O	60	60	61	67	69	73	73	67
		99.08	99.08	C_6_H_12_O_2_ + H_3_O^+^ → **C**_**6**_ **H**_**11**_ **O** ^**+**^+ 2H_2_O	0	6	11	19	23	21	14	.
		71.09	71.08	C_6_H_12_O_2_ + H_3_O^+^ → **C**_**5**_ **H**_**11**_^**+**^+ H_2_O + HCOOH	6	9	14	15	8	−	−	−
		43.05	43.05	C_6_H_12_O_2_ + H_3_O^+^ → **C**_**3**_ **H**_**7**_^**+**^+ H_2_O + C_3_H_6_O_2_	14	17	13	−	−	−	−	−
		41.04	41.04	C_6_H_12_O_2_ + H_3_O^+^ → **C**_**3**_ **H**_**5**_^**+**^ + H_2_O + C_3_H_6_O_2_ + H_2_	12	7	−	−	−	−	−	−
		29.02	29.04	C_6_H_12_O_2_ + H_3_O^+^ → **C**_**2**_ **H**_**5**_^**+**^+ H_2_O + C_4_H_8_O_2_	8	−	−	−	−	−	−	−
Decanal	No^+^	191.16	191.16	C_10_H_20_O + 2H_2_O + NO^+^ → **C**_**10**_ **H**_**19**_ **O·2H**_**2**_ **O** ^**+**^+HNO	−	−	−	−	−	−	−	10
		173.16	173.15	C_10_H_20_O + H_2_O + NO^+^ → **C**_**10**_ **H**_**19**_ **O·H**_**2**_ **O** ^**+**^+HNO	−	−	−	−	−	4	9	22
		155.14	155.14	C_10_H_20_O + NO^+^ → **C**_**10**_ **H**_**19**_ **O** ^**+**^+HNO	13	52	59	91	94	90	86	63
		137.13	137.13	C_10_H_20_O + NO^+^ → **C**_**10**_ **H**_**17**_^**+**^+HNO+ H_2_O	8	10	10	9	6	5	5	4
		95.09	95.08	C_10_H_20_O + NO^+^ → **C**_**7**_ **H**_**11**_^**+**^+C_3_H_7_O + H_2_ + NO	15	12	7	−	−	−	−	−
		81.08	81.07	C_10_H_20_O + NO^+^ → **C**_**6**_ **H**_**9**_^**+**^+C_4_H_9_O + H_2_ + NO	13	10	6	−	−	−	−	−
		67.05	67.05	C_10_H_20_O + NO^+^ → **C**_**5**_ **H**_**7**_^**+**^+C_5_H_11_O + H_2_ + NO	5	−	−	−	−	−	−	−
		57.07	57.07	C_10_H_20_O + NO^+^ → **C**_**4**_ **H**_**9**_^**+**^+C_6_H_11_O + NO	8	11	8	−	−	−	−	−
		43.04	43.05	C_10_H_20_O + NO^+^ → **C**_**3**_ **H**_**7**_^**+**^+C_7_H_11_O + H_2_ + NO	9	13	9	−	−	−	−	−
		41.03	41.04	C_10_H_20_O + NO^+^ → **C**_**3**_ **H**_**5**_^**+**^+C_7_H_11_O + 2H_2_ + NO	19	12	−	−	−	−	−	−
		39.02	39.02	C_10_H_20_O + NO^+^ → **C**_**3**_ **H**_**3**_^**+**^+C_7_H_11_O + 3H_2_ + NO	9	−	−	−	−	−	−	−
Ethyl‐phenol	O_2_^+^	123.08	123.08	C_8_H_10_O+ H_3_O ^+^(H_2_O) → C_8_H_10_O·H_3_O^+^+ O_2_ C_8_H_10_O·H_3_O^+^+ M → C_**8**_ **H**_**11**_ **O** ^**+**^+ H_2_O + M	−	−	5	7	9	12	16	27
		122.06	122.07	C_8_H_10_O + O_2_^+^ → **C**_**8**_ **H**_**10**_ **O** ^**+**^+ O_2_	22	30	32	33	34	34	34	31
		107.05	107.06	C_8_H_10_O + O_2_^+^ → **C**_**7**_ **H**_**8**_ **O** ^**+**^+ HCOOH	78	70	62	59	56	53	50	42

### 3.2 Fatty acid reactivity using H_3_O^+^ as primary ion

Fragmentation patterns were determined under humid air conditions for butanoic, pentanoic, and hexanoic acid. Reactivity of fatty acids with H_3_O^+^ has already been described under high reduced electric field conditions.^20^ Main products of the reaction were the protonated ion [M + H]^+^, along with the acylium ion [M − OH]^+^. Minor amounts of water adduct were also detected. Our result show that in the mid‐to‐high reduced electric field range (E/N = 96–144 Td), reactivity can be relatively complex, generating several alkyl fragments along with the previously mentioned acylium ion. At values of E/N of 84 Td or lower, ion chemistry is simpler, and only the acylium ion, the protonated ion, and the water adduct are observed. Reactivity is described in detail in the case of hexanoic acid (Table 1), and analogous behavior is observed for butanoic and pentanoic acid. Figure 1 shows how at E/N = 84 Td, the best compromise conditions were obtained, with minimized adduct and fragment formation and 65% to 73% protonated ion relative abundance. Similarities were observed between PTR‐MS and SIFT‐MS chemistry with the latter also being characterized by protonation and water removal as preferred reaction channels for butanoic and pentanoic acid.^21^ The perusal of an up‐to‐date SIFT‐MS Profile 3 instrument kinetic library (unpublished results) reveals that water adducts are also encountered among the products of the reaction between H_3_O^+^and fatty acids.

### 3.3 Phenol reactivity using H_3_O^+^, NO^+^, and O_2_^+^

**Figure 2:**
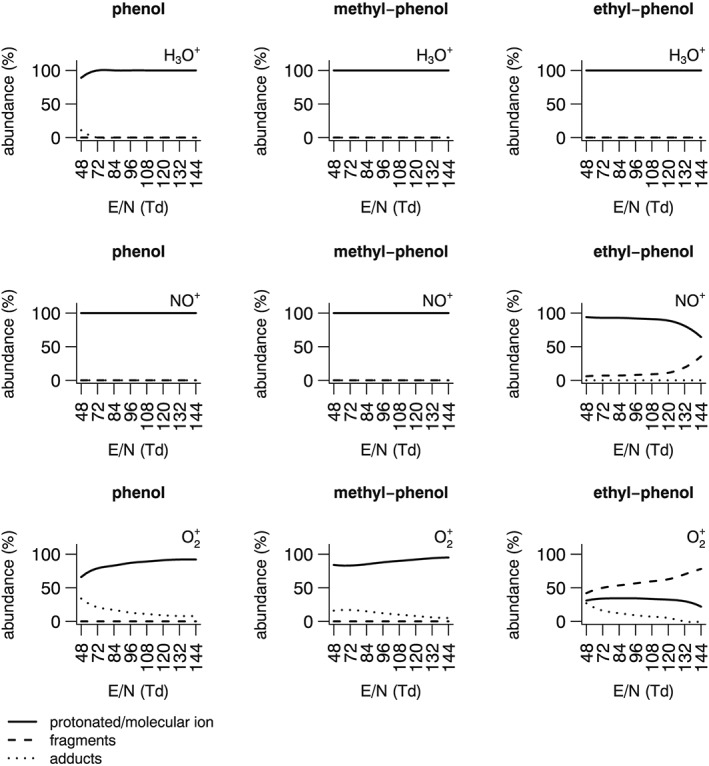
Fragmentation patterns of phenols in humid air using 3 ions

Fragmentation patterns were determined under humid air conditions for phenol, 4‐methyl‐phenol, and 4‐ethyl‐phenol. The reactivity of these phenols with the 3 ions has previously been reported using SIFT‐MS.^22^ With H_3_O^+^, reaction channels are proton transfer and water adduct formation, whereas using NO^+^ or O_2_^+^, electron transfer is observed as main reaction channel, with consequent generation of the molecular ion M^+^. O_2_^+^ ionization is known to be more energetic, and expectedly the production of a fragment of mass [M − CH_2_]^+^ is observed for ethyl‐phenol. This work represents the first description of phenol reactivity with PTR‐MS. Using H_3_O^+^, reaction chemistry is extremely simple: the protonated ion is the only detected reaction product, with the only exception represented by phenol at E/N = 48 Td, where limited quantities of a water adduct are observed (Figure 2). Similarly to SIFT‐MS, reaction with NO^+^ results into the production of a molecular ion by charge transfer. PTR‐MS reactivity is slightly more energetic, with the generation of detectable amounts of a [M − CH_2_]^+^ fragment for ethyl‐phenol. When employing O_2_^+^ as primary ion, fragmentation of ethyl‐phenol is higher, with 48% to 72% relative abundance depending upon reduced electric field conditions (Table 1). It is also worth mentioning that, when using a O_2_^+^ as primary ion, a product with mass M + 1 is observed with the 3 phenols; this can derive from the ^13^C isotopologue of the M^+^ ion, but its relative intensity exceeds the expected abundance of ^13^C. It can be hypothesized that this mass peak comes from the overlap of 2 reaction products: the ^13^C isotopologue and a protonated ion. The latter could be originating from a ligand switching reaction from a water cluster, taking place when water vapour is present within the drift tube. This is followed by a dissociative collision, made possible by the presence of a neutral species M (eg, N_2_) acting as a third body (possible reaction mechanism reported for ethyl‐phenol in Table 2). A similar mechanism was previously hypothesized for PTR‐MS, when employing H_3_O^+^/O_2_^+^ mixed ionization mode.^23^ The presence of this reaction channel must be considered when using O_2_^+^ to measure phenols at low E/N values in water‐rich matrices; its contribution is estimated as follows:

(1) $${\left[M+1\right]}_{\text{corr}}={\left[M+1\right]}_{\text{meas}}-{\left[M+1\right]}_{13C}$$

where the relative abundance of the protonated ion is obtained by subtracting the expected contribution of the ^13^C isotopologue from the measured intensity of the [M + 1]^+^ mass peak. Based on Equation (1), the reaction channel leading to the protonated ion becomes quite well represented at low E/N values, reaching 17% to 34% relative abundance.

**Table 2:** Accuracy and repeatability. Diffusion rates are evaluated using PTR‐ToF‐MS and a gravimetric measurement, using the latter as reference (*n* = 3)

		Diffusion Rate, ng min^−1^ a		
Compound	Primary Ion	Gravimetric	PTR‐ToF‐MS	Error %
Butanoic acid	H_3_O^+^	9061(±291)	8540(±543)	−5.7
Pentanoic acid	H_3_O^+^	4261(±75)	4076(±152)	4.3
Hexanoic acid	H_3_O^+^	3820(±116)	3870(±135)	−1.3
Butanal	NO^+^	39628(±922)	38112(±2692)	3.8
Decanal	NO^+^	3969(±193)	3649(±175)	8.0
Phenol	H_3_O^+^	2603(±108)	2131(±144)	18.1
	NO^+^	2565(±62)	2365(±112)	7.7
	O_2_^+^	2579(±71)	2434(±144)	5.6

a Mean(±SD).

### 3.4 Other compound/primary ion combinations

The study of the ionization of aldehydes with H_3_O^+^ and O_2_^+^ and fatty acids with NO^+^ and O_2_^+^ as primary ions provides interesting insight on PTR‐MS chemistry. When using O_2_^+^, due to the high energetic conditions typical of this ionization mode, high fragmentation was observed, especially when working at E/N = 120–144 Td. Under such conditions, no molecular ion was obtained from fatty acids, with fragments being generated at *m/z* 41, 43, and 71. At lower E/N values, the generation of a protonated ion [M + H]^+^ was observed. It is possible that this product is obtained from a reaction channel analogous to the one hypothesized for phenols. A similar situation was observed for aldehydes, with high fragmentation at high E/N (eg, less than 5% [M − H]^+^ ion with decanal at E/N = 144 Td) and [M + H]^+^ ion as main product at low E/N values. The investigation of aldehyde chemistry with H_3_O^+^ and at low E/N values revealed that―as opposed to reported aldehyde reactivity under more energetic conditions [5]―it was possible to generate stable aldehyde protonated ions. For instance, at E/N = 84 Td, the mass peak with *m/z* = 157 accounted for more than 95% of the overall signal of the decanal mass spectrum (decanal M_r_ = 156 Da). The investigation of the reactivity of fatty acids at low E/N disclosed a reaction channel leading to the formation of a nitrosonium adduct ion [M + NO]^+^ for all 3 investigated compounds. This increased in relative abundance with the reduction of drift voltage, reaching 72% to 74% at E/N = 48 Td with all 3 fatty acids. This is of undeniable interest, as it makes possible to detect all 13 compounds using the same primary ion (ie, NO^+^) exploiting a distinct reaction channel for each class: hydride abstraction for aldehydes, electron transfer for phenols, and adduct formation for fatty acids, respectively.

### 3.5 Effect of drift voltage on sensitivity and comparison between dry and humid conditions

**Figure 3:**
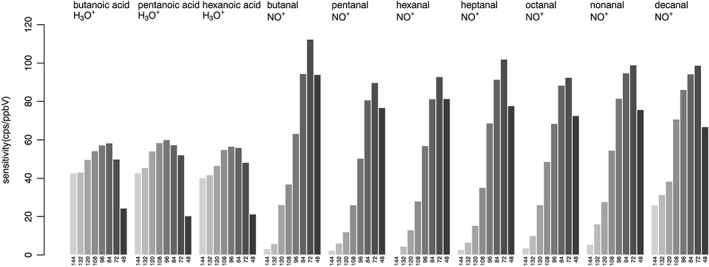
Ionization of fatty acids and aldehydes in humid air using H_3_O^+^ and NO^+^ as primary ions, respectively. Sensitivity as a function of E/N

**Figure 4:**
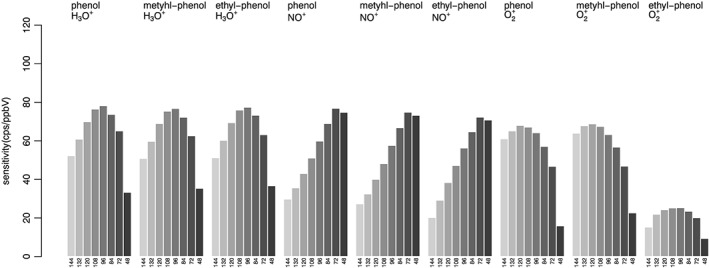
Ionization of in humid air using 3 ions. Sensitivity as a function of E/N

The choice of the optimal conditions in terms of E/N should aim to the generation of a single product ion from each compound/primary ion combination. In order to increase the informational content of the mass spectrum and to minimize overlaps, the product ion should ideally be a molecular ion generated by charge transfer, a quasi‐molecular ion originating from hydride abstraction reactions, or a protonated ion. Conditions of reduced drift voltage result in increased sensitivity, thanks to the reduced fragmentation, but also due to the increase in drift time. Sensitivity was calculated in terms of ratio between the signal (expressed in counts per second of the reference mass peak) and the concentration (expressed in ppbV and calculated considering all product ions). Results obtained for fatty acids and aldehydes (with H_3_O^+^ and NO^+^) and phenols (with the 3 ions) are shown in Figures 3 and 4, respectively. For fatty acid detection with H_3_O^+^, optimum sensitivity was obtained at E/N = 84–96 Td, with a moderate but significant improvement in comparison to more energetic conditions. In the case of aldehyde detection using NO^+^, the highest sensitivity was obtained at E/N = 72 Td, with a remarkable improvement guaranteed by the reduction in drift voltage. This was particularly evident in the case of short‐chain aldehydes: for example, butanal was detected with a sensitivity of 3 to 26 cps/ppbV for an E/N in the range 120 to 144 Td, whereas this increased to 112 cps/ppbV at E/N = 72 Td. The impact of drift voltage on sensitivity in phenol detection was evaluated with all 3 ions. Optimal sensitivity was observed with H_3_O^+^ at E/N = 96 Td. The most remarkable improvement was observed using NO^+^, with a 2‐fold increase obtained at E/N = 72 Td, compared with typical E/N = 120–144 Td. As for ionization with O_2_^+^, the generation of protonated ions at low E/N counteracted the benefit deriving from the increased drift times; maximal sensitivity was obtained at E/N = 120 Td for phenol and methyl‐phenol, whereas a moderate increased in sensitivity was observed for ethyl‐phenol at E/N = 96 Td, thanks to the reduced fragmentation guaranteed by said conditions.

Our choice of reaction conditions (water saturated air at 37°C) derives by the interest in achieving results directly applicable to breath analysis. The impact of water was further studied in some selected case studies and by inserting an in‐house developed CaCl_2_ cartridge: this was placed right before the inlet during a measurement, allowing to directly assess the impact of water removal. All these experiments were carried out at a single value of E/N = 84 Td. The impact of water on fragmentation was tested for all aldehydes with NO^+^ as primary ion: the switch from humid to dry air resulted in no effect for nonanal and decanal, while for shorter chain aldehydes, a moderate but measurable increase in fragmentation was observed (up to 9% with butanal). The switch from humid to dry air showed no measurable impact on fatty acids (H_3_O^+^ as primary ion) and phenols (NO^+^ as primary ion).

### 3.6 Comparison with a gravimetric measurement

The final step of the workflow consisted of an experiment aimed at evaluating quantitation accuracy. Several approaches are possible in terms of what is chosen as reference method. The use of diffusion tubes offers several advantages, as experiments are simple to set up and allow for direct comparison with a gravimetric standard, which represents a reliable reference, provided analyte losses can be assessed accurately. The choice of measurement conditions (60°C under nitrogen flow) was justified by the practical need to obtain a measurable weight loss in a reduced amount of time, at the same time avoiding oxidative breakdown of the compounds of choice, which could be observed for aldehydes when using air. A subset of compound/primary ion combinations was chosen: butanal, decanal (NO^+^), butanoic, pentanoic, hexanoic acid (H_3_O^+^), and phenol (all ions). Diffusion rates were estimated by means of PTR‐ToF‐MS and by measuring tube weight loss: for each of the tested compounds, it was possible to define suitable conditions whereby diffusion rates estimated with PTR‐ToF‐MS were within 8% of values extrapolated from gravimetric measurements (Table 2). The procedure showed good repeatability, as coefficients of variability in the assessment of diffusion rates were below 7%. The proposed protocol provides accuracy values similar to those reported using gas calibration mixtures,^6^ with the advantage of being applicable to any compound for which a pure standard is available.

## 4 CONCLUSION

This work outlines an experimental workflow for the reliable detection and quantification of target compounds by PTR‐MS. Based on this original workflow, we recommend researchers to approach the problem by performing a thorough screening of reduced drift field conditions with different primary ions, also testing E/N value below 120 Td, which are in most cases neglected. As demonstrated by our results, this strategy allowed to define an optimal range of reduced electric field (E/N = 72–96 Td) for the detection and quantification of aliphatic aldehydes, with a 3‐fold increase in sensitivity compared with conditions reported in the literature.^5^, ^18^ Quite interestingly, the use of low reduced drift field also disclosed surprising similarities between PTR‐MS and SIFT‐MS chemistry. SIFT‐MS is known to afford lower fragmentation than PTR‐MS due its less energetic ionization conditions: the findings reported in this work suggest that, by means of the choice of appropriate ionization settings, suitable conditions can be found where the wealth of information available on SIFT‐MS fragmentation patterns of VOCs (the so‐called “kinetic libraries”) might also be used to provide useful indications for the interpretation of PTR‐MS spectra. As additional step of the workflow, we suggest evaluating the impact of a change in humidity on VOC fragmentation patterns. This experiment is of particular interest for those fields of research (eg, breath analysis, atmospheric chemistry) where a variation in relative humidity of the matrix can affect the results. Finally, we provide a set of all‐purpose experimental conditions for gravimetric calibration, providing a quantitation accuracy within 8% of the reference standard. The use of gravimetric calibration provides an undeniable advantage over other available methods (eg, gas calibration mixtures and DSI) as it is applicable to any VOC that is readily available as authentic standard.

## Supporting information


**Table S1**. Reaction rate coefficients (k) between fatty acids, aldehydes and phenols and the three ions with E/N = 84 Td.
