# Supplementary material for: Identification and quantification of VOCs by proton transfer reaction time of flight mass spectrometry: An experimental workflow for the optimization of specificity, sensitivity, and accuracy
Source: J Mass Spectrom. 2018 Feb 21;53(4):287–95. doi: 10.1002/jms.4063 (PMC5838793; doi:10.1002/jms.4063)
Supplement: Supplementary file 1 — Table S1. Reaction rate coefficients (k) between fatty acids, aldehydes and phenols and the three ions with E/N = 84 Td. [file JMS-53-287-s001.docx]

| **Primary ion** | **Primary ion mobility** | **Neutral compound** | **Molar mass** | **Polarisability** | **Dipole moment** | **k** |
| --- | --- | --- | --- | --- | --- | --- |
|  | (cm^2^ V^-1^ s) |  | (Da) | (10^-24^cm^3^) | (Debye) | (10^-9^cm^3^ s^-1^) |
| NO^+^ | 2.91^a^ | butanal | 72.05751 | 8.22^c^ | 2.72 ^c^ | 2.71 |
| NO^+^ | 2.91 | pentanal | 86.07316 | 10.01 ^c^ | 2.79 ^c^ | 2.79 |
| NO^+^ | 2.91 | hexanal | 100.0888 | 11.78 ^c^ | 2.94 ^c^ | 2.91 |
| NO^+^ | 2.91 | heptanal | 114.1045 | 13.52 ^c^ | 2.38 ^c^ | 2.61 |
| NO^+^ | 2.91 | octanal | 128.1201 | 15.48 ^c^ | 2.54 ^c^ | 2.74 |
| NO^+^ | 2.91 | nonanal | 142.1358 | 17.41 ^c^ | 2.84 ^c^ | 2.96 |
| NO^+^ | 2.91 | decanal | 156.1514 | 19.2 ^c^ | 2.5 ^c^ | 2.76 |
| NO^+^ | 2.91 | phenol | 94.04186 | 11.18 ^c^ | 1.27 ^c^ | 1.87 |
| NO^+^ | 2.91 | methyl-phenol | 108.0575 | 13.89 ^c^ | 1.3 ^c^ | 2.01 |
| NO^+^ | 2.91 | ethyl-phenol | 122.0732 | 13 ^d^ | 1.5 ^d^ | 2.01 |
| H_3_O^+^ | 2.76 ^b^ | butanoic acid | 88.05243 | 10^e^ | 1.8^e^ | 2.67 |
| (H_2_O)H_3_O^+^ | 2.28 ^b^ | butanoic acid | 88.05243 | 10 | 1.8 | 2.11 |
| H_3_O^+^ | 2.76 | pentanoic acid | 102.0681 | 11^e^ | 1.8^e^ | 2.68 |
| (H_2_O)H_3_O^+^ | 2.28 | pentanoic acid | 102.0681 | 11 | 1.8 | 2.11 |
| H_3_O^+^ | 2.76 | hexanoic acid | 116.0837 | 12.496 | 1.572 | 2.53 |
| (H_2_O)H_3_O^+^ | 2.28 | hexanoic acid | 116.0837 | 12.496 | 1.572 | 1.97 |
| H_3_O^+^ | 2.76 | phenol | 94.04186 | 11.18 | 1.27 | 2.29 |
| (H_2_O)H_3_O^+^ | 2.28 | phenol | 94.04186 | 11.18 | 1.27 | 1.8 |
| H_3_O^+^ | 2.76 | methyl-phenol | 108.0575 | 13.89 | 1.3 | 2.46 |
| (H_2_O)H_3_O^+^ | 2.28 | methyl-phenol | 108.0575 | 13.89 | 1.3 | 1.91 |
| H_3_O^+^ | 2.76 | ethyl-phenol | 122.0732 | 13 | 1.5 | 2.49 |
| (H_2_O)H_3_O^+^ | 2.28 | ethyl-phenol | 122.0732 | 13 | 1.5 | 1.94 |
| O_2_^+^ | 2.52 | phenol | 94.04186 | 11.18 | 1.27 | 1.87 |
| O_2_^+^ | 2.52 | methyl-phenol | 108.0575 | 13.89 | 1.3 | 1.99 |
| O_2_^+^ | 2.52 | ethyl-phenol | 122.0732 | 13 | 1.5 | 2.02 |

**Table S1.** Reaction rate coefficients (k) between fatty acids, aldehydes and phenols and the three ions with E/N = 84 Td.

| **References** |
| --- |
| (a) Viehland, L.A., Mason, E.A.: Transport Properties of Gaseous Ions over a Wide Energy Range, IV. Atomic Data and Nuclear Data Tables. 60, 37–95 (1995). |
| (b) Dotan, I., Albritton, D.L., Lindinger, W., Pahl, M.: Mobilities of CO _2_ ^+^ , N _2_ H ^+^ , H _3_ O ^+^ , H _3_ O ^+^ ⋅H _2_ O, and H _3_ O ^+^ ⋅ (H _2_ O) _2_ ions in N _2_. The Journal of Chemical Physics. 65, 5028–5030 (1976). |
| (c) Cappellin, L., Karl, T., Probst, M., Ismailova, O., Winkler, P.M., Soukoulis, C., Aprea, E., Märk, T.D., Gasperi, F., Biasioli, F.: On Quantitative Determination of Volatile Organic Compound Concentrations Using Proton Transfer Reaction Time-of-Flight Mass Spectrometry. Environmental Science & Technology. 46, 2283–2290 (2012). doi:10.1021/es203985t |
| (d) Ŝpaněl, P., Smith, D.: SIFT studies of the reactions of H3O+, NO+ and O+2 with a series of volatile carboxylic acids and esters. International Journal of Mass Spectrometry and Ion Processes. 172, 137–147 (1998). |
| (e) Wang, T., Španěl, P., Smith, D.: A selected ion flow tube study of the reactions of H3O+, NO+ and O2+• with some phenols, phenyl alcohols and cyclic carbonyl compounds in support of SIFT-MS and PTR-MS. International Journal of Mass Spectrometry. 239, 139–146 (2004). |
| (f) NIST Computational Chemistry Comparison and Benchmark Database, http://cccbdb.nist.gov/ (accessed September 22, 2017). |
